# Awareness of Stroke Symptoms, Risk Factors, and Utilization of Neuroradiology Services Among the General Public in Saudi Arabia

**DOI:** 10.3390/healthcare14101410

**Published:** 2026-05-20

**Authors:** Basem Hasan Bahakeem

**Affiliations:** Department of Medicine, Umm Al-Qura University, Makkah 24381, Saudi Arabia; bhbahakeem@uqu.edu.sa

**Keywords:** neuroradiology, risk factor, Saudi Arabia, service, stroke

## Abstract

**Background:** Stroke is a major global health issue. It is among the leading causes of disability and mortality worldwide. Early stroke detection and treatment are significant in enhancing long-term outcomes. Awareness of neuroimaging is also essential because neuroimaging must be completed urgently within a limited time to diagnose and treat stroke patients correctly. This study aims to investigate awareness of stroke symptoms, risk factors, and utilization of neuroradiology services among the public in Saudi Arabia. **Methods:** This is an online survey study that was conducted in Saudi Arabia using social media platforms between January to February 2026. The questionnaire tool for this study was adapted from previous research and examined stroke awareness, symptoms, and risk factors. In addition, it examined neuroradiology awareness and utilization. The multivariable logistic regression analysis was used to identify predictors of better awareness of stroke. **Results:** A total of 415 participants were involved in this study. Around 46.7% of them were aged 25–34 years. Females formed the majority of the study sample, comprising 76.4%. Bachelor’s degree holders formed 61.4% of the study sample. Around 42.9% of the study sample were unemployed. Married participants contributed 64.3%. Almost half of the study sample (47.2%) reported that their monthly income is less than 5000 SAR. In this study, the participants demonstrated a moderate level of knowledge of stroke. The majority of the participants (70.8%) reported that they are aware of radiology centers near them that they can refer to in case of stroke emergency or follow-up, and 79.0% reported that they think that radiological imaging is important for diagnosing and treating stroke. The majority of the participants (72.3%) reported that they have heard of interventional radiology procedures for stroke. Participants aged 35–44 years and 55–64 years were less likely to have better knowledge of stroke compared to others (aOR: 0.21 (CI: 0.06–0.83); *p*-value: 0.026) and aOR: 0.17 (CI: 0.04–0.81); *p*-value: 0.026, respectively). Furthermore, participants who reported that their income level is 5000–9999 SAR and 10,000–14,999 SAR were less likely to have better knowledge of stroke compared to others (aOR: 0.32 (CI: 0.13–0.80); *p*-value: 0.014 and aOR: 0.21 (CI: 0.09–0.53); *p*-value: <0.001), respectively). On the other hand, participants who are unemployed were more likely to have better knowledge of stroke compared to others (aOR: 3.63 (CI: 1.09–12.05); *p*-value: 0.035). **Conclusions:** The current investigation demonstrated a moderate level of knowledge about strokes among the public in Saudi Arabia. Targeted interventions are mandated to improve the level of awareness about strokes, with a focus on knowledge of the correct emergency response, specifically calling an ambulance.

## 1. Introduction

Stroke is a major global health issue. It is among the leading causes of disability and mortality worldwide. In addition, it imposes a substantial burden on the community and healthcare systems [1,2]. According to a global estimation in 2021, nearly 94 million individuals live with stroke, and more than 7 million deaths are related to stroke [3]. In Saudi Arabia, stroke is the second leading cause of death, responsible for more than 10% of total mortality in the country [4], with an expectation of an increase in this mortality rate in the coming years [5]. Similarly, there is an increase in stroke risk and incidence in Saudi Arabia as a result of an increase in risk factors (such as diabetes mellitus and hypertension), changes in lifestyle, and demographic shifts [5,6].

Early stroke detection and treatment are significant in enhancing long-term outcomes, increasing survival rates, and decreasing brain damage [7]. Although stroke treatment has advanced considerably, most stroke patients from France [8], the United Kingdom (UK) [9], the United States (US) [10], and Saudi Arabia [11] delay seeking medical services. The literature shows that this delay in seeking medical services for stroke patients is linked with a lack of recognition of the earlier stroke symptoms and signs [12]. Indeed, stroke symptoms include sudden severe headache, vision changes, loss of body balance or issues in walking, speech difficulty, confusion, and weakness or numbness on one side of the body [13]. Prior studies related to stroke patients in Saudi Arabia found that delays in admission to emergency departments and insufficient symptom awareness affect optimal stroke treatment [14,15,16]. On the other hand, earlier recognition of the stroke symptoms and signs is associated with better patient outcomes [17]. Therefore, awareness of stroke symptoms and signs is critical.

Awareness of stroke risk factors is also essential for improving patient outcomes and preventing stroke. Research has shown that appropriate knowledge of stroke risk factors can prevent approximately 80% of cases [18]. Similarly, it can enhance survival rate and stroke prognoses and lead to seeking medical services earlier [19]. This prevention can be obtained by focusing on modifiable risk factors of stroke [20], including hyperchloremia, excessive alcohol drinking, heart issues, physical inactivity, unhealthy diet, obesity, diabetes, cigarette smoking, and hypertension [21,22,23].

Finally, awareness of neuroimaging is also essential because neuroimaging must be completed urgently within a limited time to diagnose and treat stroke patients correctly. Public awareness of neuroimaging services is important to examine their timely care-seeking behavior and health system literacy due to geographic variation in stroke healthcare centers. However, no prior studies have investigated public awareness about neuroimaging for stroke. This study seeks to investigate awareness of stroke symptoms, risk factors, and utilization of neuroradiology services among the public in Saudi Arabia. The findings of this study may help reduce the stroke burden and improve patient outcomes.

## 2. Methods

### 2.1. Study Design

This is an online survey study that was conducted in Saudi Arabia using social media platforms between January and February 2026.

### 2.2. Study Population and Sampling Technique

Adult individuals who are aged 18 years and older and currently living in Saudi Arabia formed the study population. The convenience sampling technique was used to recruit the study participants for this research. Participants were invited using social media platforms (X, Instagram, Facebook, and WhatsApp). The invitation letter highlighted the study’s inclusion criteria for them before participation.

### 2.3. Questionnaire Tool

The questionnaire tool for this study was adapted from previous research by Abdo Ahmed et al. [24]. The questionnaire tool comprised three sections. Sociodemographic information included age group, sex, highest level of education completed, employment status, marital status, monthly household income (which was further categorized into three socioeconomic levels based on the General Authority for Statistics in Saudi Arabia as low-income (less than 5000 SAR), middle-income (5000–14,999 SAR), and high-income categories (15,000 SAR and above) [25], region of residence, whether they live in an urban or rural area, and whether they have ever been diagnosed with stroke). There was also a Stroke Awareness, Symptoms, and Risk Factors section (whether they have heard about the term stroke before, whether they know anyone who has had a stroke, identifying warning signs and symptoms of stroke, identifying risk factors of stroke, whether they think stroke requires prompt treatment, and if someone shows signs and symptoms of stroke, what they think they should do first). Finally, there was a Neuroradiology Awareness and Utilization section (whether they have ever personally experienced symptoms that might indicate a stroke, where they sought medical care, whether they know that stroke treatment is most effective within the first few hours, whether the healthcare providers performed radiological imaging for them, what type of radiological imaging was performed, whether they are aware of radiology centers near them that they can go to in case of stroke emergency or follow-up, whether they are concerned about the safety of radiology dye (contrast agents) used in imaging tests, whether they think radiological imaging (CT/MRI) is important for diagnosing and treating (e.g., guiding clot removal or procedures) stroke, whether they have ever heard of interventional radiology procedures for stroke (e.g., catheter-based clot removal), what they think the barriers to using radiology for stroke care are, whether they think making radiology services more available in hospitals can improve stroke outcomes, if they or someone they know had stroke symptoms, how likely are they to request radiology tests (CT/MRI) as part of urgent care, and whether they have ever heard of artificial intelligence or advanced imaging being used in stroke diagnosis). The stroke knowledge score was estimated by examining participants’ knowledge of stroke warning signs and symptoms, risk factors, whether it requires prompt treatment, and the first action that should be taken if someone shows signs and symptoms of stroke. For each right answer, the participants were given a score of 1, with a maximum attainable score of 21; see the Supplementary Materials.

### 2.4. Sample Size

Using the formula n = Z^2^ × *p*(1 − *p*)/d^2^, the minimum required sample size was 384 participants, with a confidence interval of 95%, a standard deviation of 0.5, and a margin of error of 5% [26].

Z = 1.96 for 95% confidence interval, *p* = 0.5 (an assumed population proportion), and d = 0.05.

### 2.5. Ethical Approval

This research was approved by the Biomedical Research Ethics Committee at Umm Al-Qura University, Saudi Arabia (approval number HAPO-02-K-012-2025-12-3143) (date: 20 December 2025). All participants provided their informed consent before participating in this study.

### 2.6. Statistical Analysis

The Statistical Package for Social Science Software (SPSS), version 31, IBM, Armonk, NY, USA, was used to analyze the data for this research. Categorical variables were presented as frequencies and percentages. Continuous variables were presented as mean and standard deviation. The multivariable logistic regression analysis was used to identify predictors of better awareness of stroke. The findings were presented as adjusted odds ratio (aOR) with their 95% confidence interval. The significance level was assigned as *p*-value less than 0.05. Sensitivity analysis was conducted excluding participants working in the medical field and those who have personally experienced symptoms that might indicate a stroke, in order to ensure the robustness of the findings and reflect the general public more precisely.

## 3. Results

### 3.1. Sociodemographic Information

A total of 415 participants were involved in this study. Around 46.7% of them were aged 25–34 years. Females formed the majority of the study sample comprising 76.4%. Bachelor’s degree holders formed 61.4% of the study sample. Around 42.9% of the study sample were unemployed. Married participants contributed 64.3%. Almost half of the study sample (47.2%) reported that their monthly income is less than 5000 SAR. More than half of them (62.2%) reported that they live in rural areas. Gastrointestinal tract diseases, diabetes mellitus, and eye diseases were the most commonly reported comorbidities among the study participants (Table 1).

### 3.2. Knowledge of Stroke Symptoms and Risk Factors

Overall, the study participants showed low to moderate levels of knowledge concerning stroke symptoms and risk factors. The majority of the participants (90.6%) reported that they have heard about the term stroke. Around 76.6% reported that they know someone who has had a stroke. Sudden confusion, trouble speaking, or understanding speech (82.2%), sudden numbness or weakness of face, arm, or leg (77.3%), and sudden trouble walking, dizziness, loss of balance, or coordination (74.2%) were the most commonly reported correct warning signs and symptoms of stroke. On the other hand, sudden vomiting was incorrectly identified as a warning sign and symptom of stroke by 29.4% (Table 2).

The most commonly identified correct risk factors of stroke were high blood pressure, heart disease, and smoking, which accounted for 81.7%, 75.2%, and 73.0%, respectively. On the other hand, cough was mistakenly identified as a risk factor for stroke by 21.4%. The majority of the participants (72.5%) correctly identified that stroke requires prompt treatment. Only one-fifth of the study participants (20.5%) correctly identified that calling an ambulance is the first action that should be taken if someone shows signs and symptoms of stroke (Table 2).

### 3.3. Neuroradiology Awareness and Utilization

Around 33.3% (n = 138) of the participants reported that they have personally experienced symptoms that might indicate a stroke, of which 66.0% did not seek medical care. The majority of the participants (82.7%) knew that stroke treatment is most effective within the first few hours. Of those who were referred to the emergency department or outpatient clinic (n = 47), 60.7% were asked to perform radiological imaging by their healthcare providers. Ultrasound imaging was requested for 91.3% of the participants, followed by computed tomography (81.5%) (Table 3).

The majority of the participants (70.8%) reported that they are aware of radiology centers near them that they can refer to in case of stroke emergency or follow-up, and 79.0% reported that they think that radiological imaging (CT/MRI) is important for diagnosing and treating stroke. Around 35.9% of the participants reported that they are concerned about the safety of radiology dye (contrast agents) used in imaging tests. The majority of the participants (72.3%) reported that they have heard of interventional radiology procedures for stroke. The most commonly reported barriers to using radiology for stroke care were fear of radiation, cost, and availability, which accounted for 62.4%, 54.2%, and 52.3%, respectively. Around 78.5% of the participants reported that if they or someone they know had stroke symptoms, they would request radiology tests (CT/MRI) as part of their urgent care. Furthermore, only one-third of the study participants (33.3%) reported that they have heard of artificial intelligence or advanced imaging being used in stroke diagnosis (Table 3).

### 3.4. Stroke Knowledge Score Stratified by Participants’ Sociodemographic Characteristics

The sample mean knowledge score was 12.7 out of 21, indicating a moderate level of knowledge of stroke. Participants’ sociodemographic characteristics showed statistically significant difference in participants’ mean knowledge score of stroke (*p* < 0.001), except for those who reported having at least one stroke-related risk factor condition (*p*-value: 0.602) (Table 4).

### 3.5. Predictors of Better Knowledge of Stroke

The multivariable logistic regression analysis identified that participants aged 35–44 years and 55–64 years were less likely to have better knowledge of stroke compared to others. Furthermore, participants who reported that their income level is 5000–9999 SAR and 10,000–14,999 SAR (middle-income socioeconomic status category) were less likely to have better knowledge of stroke compared to others. On the other hand, participants who are unemployed were more likely to have better knowledge of stroke compared to others (aOR: 3.63 (CI: 1.09–12.05); *p*-value = 0.035), Table 5. The second model in the regression analysis was adjusted for having personally experienced symptoms that might indicate a stroke and having at least one stroke-related risk factor condition. This model identified that participants who reported having a Bachelors’ degree and those who have heard about the term stroke were more likely to have better knowledge of stroke compared to others (aOR: 10.86 (CI: 1.27–92.71); *p*-value = 0.029 and aOR: 7.23 (CI: 1.19–44.01); *p*-value = 0.032, respectively). On the other hand, participants who reported having personally experienced symptoms that might indicate a stroke were less likely to have better knowledge of stroke compared to others (aOR: 0.14 (CI: 0.03–0.68); *p*-value = 0.015) (Table 5).

Sensitivity analysis was conducted excluding participants working in the medical field and those who have personally experienced symptoms that might indicate a stroke, and demonstrated similar findings; refer to Supplementary Materials Tables S1 and S2.

## 4. Discussion

The majority of the participants reported that they have heard of interventional radiology procedures for stroke. Middle-aged participants were less likely to have better knowledge of stroke compared to others. Furthermore, middle-income participants were less likely to have better knowledge of stroke compared to others. On the other hand, participants who are unemployed were more likely to have better knowledge of stroke compared to others. The main findings of this research were as follows: the mean stroke knowledge score indicated a moderate level of knowledge about strokes. The majority of the participants reported that they are aware of radiology centers near them that they can refer to in case of stroke emergency or follow-up, and most of them reported that they think that radiological imaging is important for diagnosing and treating stroke.

In the current investigation, the mean stroke knowledge score was 12.7 out of 21, indicating a moderate level of knowledge about strokes. In addition, the most commonly identified correct risk factors of stroke were high blood pressure, heart disease, and smoking, which accounted for 81.7%, 75.2%, and 73.0%, respectively. Similarly, an earlier investigation among university students in Saudi Arabia found a moderate level of knowledge about stroke [27]. Nevertheless, previous research among the public across various countries, including Saudi Arabia [28,29], documented a low level of knowledge about stroke symptoms and/or risk factors [28,30,31], with significant differences in the level of knowledge between countries. This suggests improvement in knowledge about strokes among the Saudi public, which may result from educational initiatives [32]. The differences in level of knowledge between countries could be attributed to educational, healthcare system, and cultural factors [33]. Consistent with our findings, participants in several prior studies in Morocco [34], Lebanon [35], and Saudi Arabia [6,36] identified high blood pressure as the most common risk factor for stroke. Also, the public in India cited heart disease and smoking as common risk factors for stroke [37]. Indeed, high blood pressure is established as the most significant risk factor for stroke [38]. Identification of modifiable risk factors for stroke, such as high blood pressure, cardiac causes, and smoking, is essential to reduce stroke burden [20]. The literature documented that knowledge about stroke modifiable risk factors and symptoms is crucial for reducing mortality and morbidity related to stroke [18] and preventing delays in stroke treatment [39]. Knowledge about stroke modifiable risk factors can encourage individuals to avoid triggering factors [40]. On the other hand, poor knowledge about stroke can result in delaying treatment [41], increasing stroke risk, and many other adverse outcomes [42]. Since our study underscores a moderate level of knowledge about strokes, targeted interventions are required to increase the level of knowledge about strokes among the Saudi public.

In our study, most of the participants (72.5%) correctly identified that stroke requires prompt treatment, and 82.7% knew that stroke treatment is most effective within the first few hours. Nevertheless, only one-fifth of the participants (20.5%) correctly identified calling an ambulance as the first action that should be taken if someone shows signs and symptoms of stroke. In line with our findings, 87% of patients from Saudi Arabia [43], 85% of patients from South Asia [44], and 82% of patients from Southern India [45] correctly identified that stroke required immediate medical treatment. About 93% [46] and 87% of the public from Saudi Arabia [43] and 77% of patients from South Carolina [47] were aware that stroke is a medical emergency. Despite this high awareness, as observed among our participants, the proportion of individuals who would call an ambulance or emergency services when experiencing stroke symptoms and signs remains low in the literature (less than 70%), with variability across regions. However, all of them reported a higher proportion than that among our participants. For instance, about 64% of participants from Greece believed that stroke symptoms could improve immediately by calling an ambulance [48]. Likewise, 59% in Iran [49] stated that they would call an ambulance when experiencing a stroke. Other studies observed lower rates among their participants; only 35% in India [50], 34% in Saudi Arabia [51] and Austria, and 32% in Malaysia [31] said that they would take the same action. These findings highlight the need to improve public knowledge of the correct emergency response for stroke, specifically calling an ambulance.

Indeed, a stroke is a medical emergency that mandates prompt medical treatment [52]. Transporting patients with stroke by ambulance is essential to shorten prehospital delays and enable patients to receive the most effective treatment [53]. In contrast, delaying the transportation of patients with stroke to the emergency department is linked with severe mental and physical disabilities and mortality [5,54]. Prior studies demonstrated that several factors, including the identity of the caller, stroke recognition, clinical factors, sociodemographic elements [55], and knowledge about stroke signs and symptoms [56], may influence the timing of seeking ambulance assistance for stroke patients. Hence, it is essential to increase knowledge about stroke signs and symptoms among the Saudi public and assess the influence of other factors on their timing to call an ambulance if someone shows signs and symptoms of stroke.

In this study, among those who presented to the emergency department or outpatient clinic, 60.7% were asked by their healthcare providers to undergo radiologic imaging. Ultrasound imaging was requested for 91.3% of the participants, followed by CT (81.5%). Neuroimaging aids in guiding immediate treatment options and confirming diagnoses for stroke patients by assessing collateral blood flow, evaluating tissue viability through perfusion assessment, identifying vessel stenosis or occlusion, and ruling out acute hemorrhage [57].

Still, in clinical settings, the two most commonly used neuroimaging techniques for stroke are CT and MRI [58]. These are not aligned with our findings, as the two most commonly used imaging techniques for our participants were ultrasound and CT. These differences may result from several factors, including organizational factors, patients’ characteristics, and availability, which have been documented to affect the selection of imaging for stroke patients [59]. In line with these documented factors, the most commonly reported barriers to using radiology for stroke care among our participants were fear of radiation, cost, and availability, which accounted for 62.4%, 54.2%, and 52.3%, respectively. These barriers potentially affected both the proportion of participants asked by their healthcare providers to undergo radiologic imaging and the imaging modality selected by healthcare providers. Likewise, another prior study in Tanzania found availability, high cost, and low health literacy among other barriers to accessing stroke care services [60]. Research from Saudi Arabia reported multiple challenges in the use of MRI for stroke diagnosis; the high cost of MRI devices is the most significant, as it limits their availability in some healthcare services [30]. Neuroimaging techniques are vital for the diagnosis, treatment, prognosis, and clinical outcomes of stroke patients [61]; therefore, addressing the aforementioned barriers is essential to optimize stroke treatment and ensure that all stroke patients have equal access to healthcare services, facilities, and resources.

Despite reported barriers to using radiology for stroke care, our findings demonstrate that the public has relatively good awareness of the role of radiologic imaging in stroke care, as most of the participants (70.8%) reported that they are aware of radiology centers near them that they can refer to in case of stroke emergency or follow-up, 79.0% reported that they think that radiological imaging is important for diagnosing and treating stroke, and 72.3% reported that they have heard of interventional radiology procedures for stroke. This awareness is higher than that reported among the public in Egypt, as only about 45% of them indicate that they would visit a neurologist [62]. The awareness of the role of radiologic imaging in stroke care among the Saudi public may have a positive impact on early diagnosis and treatment for stroke, particularly if the aforementioned barriers are addressed.

In this study, participants aged 35–44 years and 55–64 years were less likely to have better knowledge of stroke compared to those in other age groups. Furthermore, participants with income levels of 5000–9999 SAR and 10,000–14,999 SAR (middle-income socioeconomic status category) were less likely to have better knowledge of stroke compared to others. The lower likelihood of having better knowledge of stroke for participants aged 35–44 years and 55–64 years may indicate that stroke-related educational interventions about stroke did not target them. In line with this, a prior study performed in Saudi Arabia shows that the lowest level of received knowledge of stroke was among the age group 35–44 years [63]. Moreover, several prior studies reported that higher income levels or economic status are linked with better stroke knowledge [64,65,66,67], which is consistent with our findings. Consequently, to enhance stroke patients’ outcomes and decrease stroke risk in Saudi Arabia, educational interventions about stroke must target all populations, with a focus on those aged 35–44 years and 55–64 years, as well as those with lower income.

On the other hand, our study revealed that unemployed participants were more likely to have better knowledge of stroke compared to others. Agreeing with our findings, an earlier investigation among the Saudi public observed a higher likelihood of having better knowledge of stroke among unemployed participants than employed participants [68]. This greater knowledge among unemployed individuals may be due to having more time to attend educational activities or to prior experience of stroke. Aligned with this, one study reported a higher stroke risk among unemployed individuals [69]. However, other studies demonstrated that unemployed individuals have low knowledge of stroke compared with employed individuals [70,71]. This discrepancy could be related to differences in cultural, educational, and socioeconomic factors.

Adjusting for having personally experienced symptoms that might indicate a stroke and having at least one stroke-related risk factor condition in the regression analysis identified that participants who reported having a Bachelors’ degree and those who have heard about the term stroke were more likely to have better knowledge of stroke compared to others, while those who reported having personally experienced symptoms that might indicate a stroke were less likely to have better knowledge of stroke compared to others. Previous studies emphasized the role of educational attainment as an important predictor of disease awareness and knowledge [35,72]. A previous study in Lebanon identified that having higher levels of education and knowing a stroke patient were predictors for recall of more symptoms and risk factors for stroke [35]. Another study from India found that better knowledge of stroke was associated with knowing someone who experienced stroke specifically if it is from the same family [73].

This study has limitations. This study did not examine the percentage distribution of the different medical professions participating in the study (physicians, nurses, etc.), as this may explain the relatively low level of correct responses. The use of convenience sampling technique might have affected the generalizability of the study findings. The use of social media platforms to recruit the study participants increased the possibility of selection bias. The relatively small sample size might have also affected the generalizability of the study findings as the study sample mainly comprised young and highly educated subjects. This research did not collect data on the geographical spread of study respondents. This is a single-country study, which limits the generalizability of its findings for other countries. The cross-sectional study design restricted the ability to examine causality across the study variables. Stroke-disease history was not examined in this research, which restricted the ability to estimate the prevalence of stroke among the study participants. Therefore, the study findings should be interpreted carefully.

## 5. Conclusions

The current investigation demonstrated a moderate level of knowledge about strokes among the public in Saudi Arabia. Targeted interventions are mandated to improve the level of awareness about strokes, with a focus on knowledge of the correct emergency response, specifically calling an ambulance. The public has relatively good awareness of the role of radiologic imaging in stroke care; however, they face many barriers to utilizing radiology for stroke care. Even healthcare providers had some deficiencies in adherence to recommended neuroimaging practices for stroke diagnosis and management. Addressing these barriers and problems is essential to optimizing stroke treatment. Finally, individuals’ age, income levels, and employment status had a significant impact on the level of knowledge about stroke. Hence, educational interventions about stroke must focus on those within the age, income level, and employment groups with a lower knowledge of stroke.

## Figures and Tables

**Table 1:** Sociodemographic information of the participants.

Sociodemographic Information	Frequency	Percentage
Age group		
18–24 years	44	10.6%
25–34 years	194	46.7%
35–44 years	65	15.7%
45–54 years	62	14.9%
55–64 years	32	7.7%
65 years and older	18	4.3%
Sex		
Females	317	76.4%
Males	98	23.6%
Highest level of education completed		
Secondary school	45	10.8%
Diploma/Technical degree	68	16.4%
Bachelor’s degree	255	61.4%
Postgraduate degree (Master’s/PhD)	33	8.0%
Primary school or lower	14	3.4%
Employment status		
Student	67	16.1%
Unemployed	178	42.9%
Retired	38	2.9%
Employed outside the medical field	87	21.0%
Employed in the medical field	45	10.8%
Marital status		
Widowed	22	5.3%
Single	65	15.7%
Married	267	64.3%
Divorced	61	14.7%
Monthly household income (Saudi riyals)		
Less than 5000 SAR	196	47.2%
5000–9999 SAR	70	16.9%
10,000–14,999 SAR	84	20.2%
15,000–19,999 SAR	43	10.4%
20,000 SAR or more	22	5.3%
Do you live in an urban or rural area?		
Urban	157	37.8%
Rural	258	62.2%
Have you ever been diagnosed with any of the following? (multiple-answer question)		
GIT diseases	62	14.9%
Diabetes mellitus	55	13.3%
Eye diseases	55	13.3%
Dyslipidemia	50	12.0%
Chronic kidney diseases	43	10.4%
Hypertension	39	9.4%
Heart disease	37	8.9%
Other CNS diseases	35	8.4%

SAR: Saudi Arabia riyal; GIT: Gastrointestinal tract; CNS: Central nervous system.

**Table 2:** Items assessing knowledge of stroke symptoms and risk factors.

Knowledge Items	Frequency	Percentage
Have you heard about the term stroke? (Yes)	376	90.6%
Do you know anyone who has had a stroke? (Yes)	318	76.6%
*Identifying Warning Signs and Symptoms of Stroke*		
Which of the following are signs and symptoms of stroke?		
Sudden confusion, trouble speaking, or understanding speech *	341	82.20%
Sudden numbness or weakness of face, arm, or leg *	321	77.30%
Sudden trouble walking, dizziness, loss of balance, or coordination *	308	74.20%
Sudden trouble seeing in one or both eyes *	301	72.50%
Sudden severe headache with no known cause *	283	68.20%
Sudden nosebleed *	90	21.70%
High temperature *	88	21.20%
Sudden vomiting	122	29.40%
*Identifying Risk Factors of Stroke*		
High blood pressure *	339	81.7%
Heart disease *	312	75.2%
Smoking *	303	73.0%
High cholesterol *	283	68.2%
Stress *	280	67.5%
Alcohol consumption *	257	61.9%
Diabetes mellitus *	255	61.4%
Family history *	136	32.8%
Unhealthy diet *	123	29.6%
Lack of exercise *	143	34.5%
Obesity or overweight *	116	28.0%
Atrial fibrillation *	98	23.6%
Cough	89	21.4%
Stroke requires prompt treatment (Yes) *	301	72.5%
If someone shows signs and symptoms of stroke, what do you think you should do first?		
Give them Aspirin	181	43.6%
Contact their family	47	11.3%
Take them to the hospital or clinic	86	20.7%
Call an ambulance *	85	20.5%
Call a healthcare provider	16	3.9%

* Right answer included in the knowledge score estimation.

**Table 3:** Items assessing neuroradiology awareness and utilization.

Neuroradiology Awareness and Utilization Items	Frequency	Percentage
Have you ever personally experienced symptoms that might indicate a stroke? (Yes)	138	33.3%
(If Yes) Where did you seek medical care? (n = 138)		
Emergency department	23	16.6%
Outpatient clinic	24	17.3%
Did not seek medical care	91	66.0%
Do you know that stroke treatment is most effective within the first few hours? (Yes)	343	82.7%
Did healthcare providers perform radiological imaging for you? (Yes) (n = 47)	29	60.7%
(If Yes) What type of radiological imaging was performed? (multiple-answer question) (n = 29)		
CT scan (computed tomography)	24	81.5%
MRI (magnetic resonance imaging)	11	38.1%
Ultrasound (carotid Doppler)	26	91.3%
Are you aware of radiology centers near you that you can refer to in case of stroke emergency or follow-up? (Yes)	294	70.8%
Are you concerned about the safety of radiology dye (contrast agents) used in imaging tests? (Yes)	149	35.9%
Do you think radiological imaging (CT/MRI) is important for diagnosing and treating (e.g., guiding clot removal or procedures) stroke? (Yes)	328	79.0%
Have you ever heard of interventional radiology procedures for stroke (e.g., catheter-based clot removal)? (Yes)	300	72.3%
What do you think are barriers to using radiology for stroke care? (multiple-answer question)		
Fear of radiation	259	62.4%
Cost	225	54.2%
Availability	217	52.3%
Lack of awareness	138	33.3%
Others	42	10.1%
Do you think making radiology services more available in hospitals can improve stroke outcomes? (Yes)	352	84.8%
If you or someone you know had stroke symptoms, how likely are you to request radiology tests (CT/MRI) as part of urgent care?		
Very likely	259	62.4%
Likely	67	16.1%
Neutral	41	9.9%
Unlikely	33	8.0%
Very unlikely	15	3.6%
Have you ever heard of artificial intelligence or advanced imaging being used in stroke diagnosis? (Yes)	140	33.7%

**Table 4:** Stroke knowledge score stratified by participants’ sociodemographic characteristics.

Sociodemographic Information	Mean Knowledge Score	*p*-Value
Age group		
18–24 years	12.4 (5.9)	<0.001
25–34 years	14.1 (2.9)	
35–44 years	10.9 (4.6)	
45–54 years	10.8 (4.5)	
55–64 years	10.7 (4.8)	
65 years and older	14.3 (5.9)	
Sex		
Females	13.2 (4.2)	<0.001
Males	11.1 (4.9)	
Highest level of education completed		
Secondary school	11.3 (5.1)	<0.001
Diploma/Technical degree	11.1 (4.7)	
Bachelor’s degree and Postgraduate degree (Master’s/PhD)	13.2 (4.0)	
Primary school or lower	14.7 (5.5)	
Employment status		
Student	11.3 (5.5)	<0.001
Unemployed	14.5 (2.4)	
Retired	11.2 (4.3)	
Employed outside the medical field	10.3 (4.6)	
Employed in the medical field	13.7 (5.4)	
Marital status		
Widowed	12.8 (4.7)	<0.001
Single	12.3 (6.2)	
Married	13.3 (3.6)	
Divorced	10.2 (4.5)	
Monthly household income (Saudi riyals)		
Less than 5000 SAR	14.4 (3.0)	<0.001
5000–9999 SAR	11.1 (4.7)	
10,000–14,999 SAR	10.3 (4.9)	
15,000–19,999 SAR	12.1 (4.9)	
20,000 SAR or more	12.9 (4.7)	
Do you live in an urban or rural area?		
Urban	11.8 (5.5)	<0.001
Rural	13.2 (3.5)	
Have you heard about the term stroke?		
No	11.5 (6.2)	0.073
Yes	12.8 (4.2)	
Do you know anyone who has had a stroke?		
No	11.0 (5.4)	<0.001
Yes	13.2 (3.9)	
Have you ever personally experienced symptoms that might indicate a stroke?		
No	12.9 (5.2)	0.008
Yes	10.2 (3.6)	
Participants who have at least one stroke-related risk factor condition (hypertension, heart disease, diabetes mellitus, or dyslipidemia).		
No	12.8 (4.1)	0.602
Yes	12.5 (5.1)	

SAR: Saudi Arabia riyal.

**Table 5:** Predictors of better knowledge of stroke.

Sociodemographic Information	Adjusted Odds Ratio with 95% Confidence Interval (Model One)	*p*-Value	Adjusted Odds Ratio with 95% Confidence Interval (Model Two)	*p*-Value
Age group				
18–24 years	Reference category		Reference category	
25–34 years	1.17 (0.35–3.90)	0.795	3.64 (0.06–231.90)	0.542
35–44 years	0.21 (0.06–0.83)	0.026 *	0.16 (0.00–8.83)	0.374
45–54 years	0.42 (0.12–1.55)	0.193	0.29 (0.01–16.25)	0.545
55–64 years	0.17 (0.04–0.81)	0.026 *	0.09 (0.00–8.04)	0.291
65 years and older	3.52 (0.70–17.6)	0.127	-	
Sex				
Females	Reference category		Reference category	
Males	0.72 (0.38–1.36)	0.315	0.91 (0.26–3.19)	0.877
Highest level of education completed				
Secondary school	Reference category		Reference category	
Diploma/Technical degree	0.60 (0.23–1.56)	0.296	2.94 (0.51–16.98)	0.227
Bachelor’s degree	2.03 (0.77–5.34)	0.154	10.86 (1.27–92.71)	0.029 *
Postgraduate degree (Master’s/PhD)	1.35 (0.37–4.92)	0.650	2.21 (0.18–27.40)	0.537
Primary school or lower	1.76 (0.37–8.32)	0.478	-	
Employment status				
Student	Reference category		Reference category	
Unemployed	3.63 (1.09–12.05)	0.035 *	1.60 (0.06–45.67)	0.785
Retired	0.81 (0.25–2.65)	0.723	1.56 (0.05–48.78)	0.799
Employed outside the medical field	0.55 (0.21–1.49)	0.241	0.75 (0.04–15.14)	0.851
Employed in the medical field	1.70 (0.58–4.96)	0.335	1.13 (0.04–32.41)	0.943
Marital status				
Widowed	Reference category		Reference category	
Single	0.52 (0.12–2.32)	0.378	0.66 (0.01–65.87)	0.860
Married	0.62 (0.18–2.11)	0.447	0.53 (0.01–19.93)	0.734
Divorced	0.45 (0.12–1.66)	0.232	0.39 (0.01–19.49)	0.639
Monthly household income (Saudi riyals)				
Less than 5000 SAR	Reference category		Reference category	
5000–9999 SAR	0.32 (0.13–0.80)	0.014 *	0.33 (0.06–1.76)	0.193
10,000–14,999 SAR	0.21 (0.09–0.53)	<0.001	0.47 (0.09–2.40)	0.365
15,000–19,999 SAR	0.62 (0.22–1.80)	0.381	0.80 (0.10–6.65)	0.837
20,000 SAR or more	0.43 (0.12–1.54)	0.194	0.25 (0.03–2.25)	0.217
Do you live in an urban or rural area?				
Urban	Reference category		Reference category	
Rural	0.55 (0.28–1.09)	0.087	0.20 (0.03–1.20)	0.078
Have you heard about the term stroke?				
No	Reference category		Reference category	
Yes	1.04 (0.42–2.57)	0.930	7.23 (1.19–44.01)	0.032 *
Do you know anyone who has had a stroke?				
No	Reference category		Reference category	
Yes	1.91 (0.99–3.71)	0.055	1.30 (0.35–4.81)	0.698
Have you ever personally experienced symptoms that might indicate a stroke?				
No			Reference category	
Yes			0.14 (0.03–0.68)	0.015 *
Participants who have at least one stroke-related risk factor condition (hypertension, heart disease, diabetes mellitus, or dyslipidemia).				
No			Reference category	
Yes			0.934 (0.29–2.97)	0.908

SAR: Saudi Arabia riyal. Model two adjusted for having personally experienced symptoms that might indicate a stroke and having at least one stroke-related risk factor condition, * *p* < 0.05.

## Data Availability

The datasets for this study are not publicly available due to patient confidentiality, but they are available from the corresponding author upon reasonable request.

## Supplementary Materials

The following supporting information can be downloaded at: https://www.mdpi.com/article/10.3390/healthcare14101410/s1, Table S1. Stroke knowledge score stratified by participants’ sociodemographic. Table S2. Predictors of better knowledge of stroke.
