# Supplementary material for: Awareness of Stroke Symptoms, Risk Factors, and Utilization of Neuroradiology Services Among the General Public in Saudi Arabia
Source: Healthcare (Basel). 2026 May 20;14(10):1410. doi: 10.3390/healthcare14101410 (PMC13205632; doi:10.3390/healthcare14101410)
Supplement: Supplementary file 1 [file healthcare-14-01410-s001.zip › healthcare-4265443-supplementary.pdf]

Supplementary material: Sensitivity analysis excluding participants working in the medical filed and those who have personally experienced symptoms that might indicate a stroke.

Table S1: Stroke knowledge score stratified by participants' sociodemographic

Sociodemographic Information	Mean knowledge score	P-value
Age group		
18-24 years	12.9 (6.1)	<0.001
25-34 years	14.3 (2.9)	
35-44 years	11.8 (5.7)	
45-54 years	10.5 (5.2)	
55-64 years	13.0 (5.5)	
65 years and older	11.1 (6.1)	
Sex		
Females	13.7 (4.2)	0.003
Males	11.6 (5.5)	
Highest level of education completed:		
Secondary school	11.4 (6.0)	<0.001
Diploma/Technical degree	11.5 (5.5)	
Bachelor’s degree	14.1 (3.5)	
Postgraduate degree (Master’s/PhD)	12.2 (6.1)	
Primary school or lower	12.6 (5.6)	
Employment status		
Student	11.1 (6.2)	<0.001
Unemployed	14.8 (2.0)	
Retired	10.9 (4.4)	
Employed outside the medical field	11.0 (5.5)	
Marital status		
Widowed	13.2 (4.5)	<0.001
Single	12.4 (6.6)	
Married	13.9 (3.3)	
Divorced	10.1 (5.9)	
Monthly household income (Saudi Riyals)		
Less than 5,000 SAR	14.6 (2.9)	<0.001
5,000 – 9,999 SAR	11.1 (5.6)	
10,000 – 14,999 SAR	10.2 (5.9)	
15,000 – 19,999 SAR	13.7 (5.3)	
20,000 SAR or more	12.3 (4.6)	
Do you live in an urban or rural area?		
Urban	11.9 (6.0)	<0.001
Rural	14.1 (3.0)	

Table S2: Predictors of better knowledge of stroke

Sociodemographic Information	Adjusted odds ratio with 95% confidence interval	P-value
Age group		
18-24 years	Reference category	

25-34 years	0.36 (0.06-2.33)	0.283
35-44 years	0.16 (0.02-1.16)	0.070
45-54 years	0.11 (0.01-0.94)	0.044*
55-64 years	0.12 (0.01-1.24)	0.075
65 years and older	0.14 (0.01-1.68)	0.120
Sex		
Females	Reference category	
Males	0.72 (0.29-1.83)	0.493
Highest level of education completed:		
Secondary school	Reference category	
Diploma/Technical degree	0.67 (0.20-2.30)	0.527
Bachelor's degree	1.71 (0.40-7.34)	0.473
Postgraduate degree (Master's/PhD)	4.31 (0.67-27.84)	0.125
Primary school or lower	0.89 (0.09-8.82)	0.923
Employment status		
Student	Reference category	
Unemployed	20.35 (2.95-140.34)	0.002**
Retired	2.77 (0.38-20.41)	0.318
Employed outside the medical field	2.61 (0.50-13.71)	0.258
Marital status		
Widowed	Reference category	
Single	1.47 (0.19-11.59)	0.718
Married	0.94 (0.16-5.64)	0.949
Divorced	0.87 (0.12-6.55)	0.890
Monthly household income (Saudi Riyals)		
Less than 5,000 SAR	Reference category	
5,000 – 9,999 SAR	0.49 (0.15-1.63)	0.245
10,000 – 14,999 SAR	0.19 (0.06-0.64)	0.007**
15,000 – 19,999 SAR	1.11 (0.27-4.62)	0.888
20,000 SAR or more	0.19 (0.04-0.98)	0.047*
Do you live in an urban or rural area?		
Urban	Reference category	
Rural	1.44 (0.56-3.73)	0.452

Using a cut-off point 13.3 as the mean knowledge score this cohort to define the dummy variable for the regression analysis.

The questionnaire tool:

Awareness of Stroke Symptoms, Risk Factors, and Utilization of Neuroradiology Services Among the General Public in Saudi Arabia

---

Section A: Sociodemographic Information

1. Age group:

☐ 18–24

☐ 25–34

☐ 35–44
☐ 45–54
☐ 55–64
☐ 65 or above
2. Gender:
☐ Male
☐ Female
3. Highest level of education completed:
☐ Primary school or lower
☐ Secondary school
☐ Diploma/Technical degree
☐ Bachelor’s degree
☐ Postgraduate degree (Master’s/PhD)
4. Employment status:
☐ Employed in the medical field
☐ Employed outside the medical field
☐ Student
☐ Retired
☐ Unemployed
5. Marital status:
☐ Single
☐ Married
☐ Divorced
☐ Widowed
6. Monthly household income (Saudi Riyals):
☐ Less than 5,000 SAR
☐ 5,000 – 9,999 SAR
☐ 10,000 – 14,999 SAR
☐ 15,000 – 19,999 SAR
☐ 20,000 SAR or more
☐ Prefer not to answer
7. Region of residence:
☐ Central region
☐ Western region
☐ Eastern region
☐ Northern region
☐ Southern region
8. Do you live in an urban or rural area?
☐ Urban
☐ Rural
9. Have you ever been diagnosed with any of the following? (check all that apply)
☐ Hypertension
☐ Diabetes
☐ Heart disease
☐ High cholesterol
☐ Chronic kidney diseases
☐ GIT Diseases
☐ Eye diseases
☐ CNS diseases
☐ Others.

Section B: Stroke Awareness, Symptoms, and Risk Factors
Q1. Have you heard about the term stroke?
☐ Yes ☐ No
Q2. Do you know anyone who has had a stroke?
☐ Yes ☐ No
Q3. Identifying Warning Signs and Symptoms of Stroke
If you think the following are signs and symptoms of stroke, please answer Yes / No / I do not know.
3a. Sudden confusion, trouble speaking, or understanding speech
☐ Yes ☐ No ☐ I do not know
3b. Sudden nosebleed
☐ Yes ☐ No ☐ I do not know
3c. Sudden numbness or weakness of face, arm, or leg
☐ Yes ☐ No ☐ I do not know
3d. Sudden trouble seeing in one or both eyes
☐ Yes ☐ No ☐ I do not know
3e. Sudden vomiting (trap question)
☐ Yes ☐ No ☐ I do not know
3f. Sudden trouble walking, dizziness, loss of balance, or coordination
☐ Yes ☐ No ☐ I do not know
3g. Sudden severe headache with no known cause
☐ Yes ☐ No ☐ I do not know
3h. High temperature
☐ Yes ☐ No ☐ I do not know
Q4. Identifying Risk Factors of Stroke
Answer Yes / No / I do not know for each.
4a. Smoking
☐ Yes ☐ No ☐ I do not know
4b. Cough (trap question)
☐ Yes ☐ No ☐ I do not know
4c. Lack of exercise
☐ Yes ☐ No ☐ I do not know
4d. High blood pressure
☐ Yes ☐ No ☐ I do not know
4e. Heart disease
☐ Yes ☐ No ☐ I do not know
4f. Family history
☐ Yes ☐ No ☐ I do not know
4g. High cholesterol
☐ Yes ☐ No ☐ I do not know
4h. Obesity or overweight
☐ Yes ☐ No ☐ I do not know
4i. Diabetes
☐ Yes ☐ No ☐ I do not know
4j. Unhealthy diet
☐ Yes ☐ No ☐ I do not know
4k. Stress
☐ Yes ☐ No ☐ I do not know
4l. Alcohol consumption

☐ Yes ☐ No ☐ I do not know
4m. Atrial fibrillation
☐ Yes ☐ No ☐ I do not know
Q5. Do you think stroke requires prompt treatment?
☐ Yes ☐ No ☐ I do not know
Q6. If someone shows signs and symptoms of stroke, what do you think you should do first? (Choose only one)
☐ Give them Aspirin
☐ Contact their family
☐ Take them to the hospital or clinic
☐ Call an ambulance (appropriate action)
☐ Call a health care provider
Section C: Neuroradiology Awareness and Utilization
1. Have you ever personally experienced symptoms that might indicate a stroke?
☐ Yes ☐ No
2. (If Yes) Where did you seek medical care?
☐ Emergency Department
☐ Outpatient Clinic
☐ Did not seek medical care
3. Do you know that stroke treatment is most effective within the first few hours?
☐ Yes ☐ No
4. Did healthcare providers perform radiological imaging for you?
☐ Yes ☐ No ☐ I do not know
5. (If Yes) What type of radiological imaging was performed?
☐ CT Scan (Computed Tomography)
☐ MRI (Magnetic Resonance Imaging)
☐ Ultrasound (Carotid Doppler)
☐ I do not know
6. Are you aware of radiology centers near you that you can go to in case of stroke emergency or follow-up?
☐ Yes ☐ No
7. Are you concerned about the safety of radiology dye (contrast agents) used in imaging tests?
☐ Yes ☐ No ☐ I do not know
8. Do you think radiological imaging (CT, MRI) is important for diagnosing and treating (e.g., guiding clot removal or procedures) stroke?
☐ Yes ☐ No ☐ I do not know
9. Have you ever heard of interventional radiology procedures for stroke (e.g., catheter-based clot removal)?
☐ Yes ☐ No
10. What do you think are barriers to using radiology for stroke care?
☐ Cost
☐ Availability
☐ Lack of awareness
☐ Fear of radiation
☐ Others
11. Do you think making radiology services more available in hospitals can improve stroke outcomes?
☐ Yes ☐ No ☐ I do not know
12. If you or someone you know had stroke symptoms, how likely are you to request radiology tests (CT/MRI) as part of urgent care?
☐ Very likely
☐ Likely

☐ Neutral
☐ Unlikely
☐ Very unlikely
13. Have you ever heard of artificial intelligence or advanced imaging being used in stroke diagnosis?
☐ Yes ☐ No
